# Perceived insufficient pedagogical content knowledge in teaching movement and physical activity. Experiences from an action-oriented study among educators in early childhood education and care

**DOI:** 10.3389/fspor.2022.1050311

**Published:** 2023-01-16

**Authors:** Ann-Christin Sollerhed

**Affiliations:** Faculty of Teacher Education, Kristianstad University, Kristianstad, Sweden

**Keywords:** movement, physical activity, early childhood, metacognition, ECEC, pedagogical content knowledge, early childhood teacher education

## Abstract

Movement and physical activity (MoPA) are critical to children's health and development. Many children aged 1–5 years are enrolled in Early Childhood Education and Care (ECEC) in Sweden, and high expectations are placed on educators to deliver education of sufficient quality to support children's development. The aim of the 18-month-long action-based study was to investigate how 88 ECEC educators in five preschools perceived and experienced the priority and teaching of MoPA. The educators planned and implemented MoPA sessions among children. They filmed sequences from the sessions, which were shown in the focus groups and were the starting point for the collegial discussions. Content analysis of the focus group discussions revealed three themes: Teaching aspects; Educational aspects; Structural aspects, with associated subthemes. During the project with the trial-and-error MoPA teaching, the educators detected insufficient PCK to teach MoPA and that teaching was often replaced with free play. Increased metacognition made the educators aware of children's different MoPA levels and that free play did not always increase all children's skills. The perceived insufficient pedagogical content knowledge to teach MoPA was perceived as a troublesome barrier for promoting MoPA. During the project, the educators' metacognition about MoPA increased, which made the educators aware of children's different MoPA levels and that free play did not always increase all children's skills. Despite of increased metacognition, most of the educators were not ready to leave their comfort zones and were not open to extra work or effort when it came to MoPA. However, the educators demonstrated the need for improved education in MoPA in early childhood teacher education, as well as the need for continuous education for working educators in ECEC to enhance the pedagogic content knowledge for adequate teaching in MoPA, which is important for children's present development and future health.

## 1. Introduction

In 2021, 86% of children of preschool age (one to five years) in Sweden participated in Early Childhood Education and Care (ECEC). On average, children spend 31 h a week in ECEC (1). Parents share the responsibility for the child's education and development with ECEC, and high expectations are placed on the educators to deliver adequate education to support children's development in all domains (2). The ECEC curriculum describe a broad range of domains which should be addressed, for example language, mathematics, science, music, health, movement (3). Educators in ECEC are predominantly generalists and not specialists in all domains, but they tend to be more likely to teach discrete skills, such as language and science among children (4) and recognize barriers to effective teaching in domains such as movement and physical activity (MoPA) (5). The educators require knowledge to make decisions about what and how to teach MoPA, for example, how to plan and perform activities with the children, detect errors and design task progressions (6). Several reports have raised the issue of the low priority placed on the domain of MoPA in ECEC (7, 8). In addition, low levels of physical activity (PA) in general are reported among young children (9, 10), which may lead to adverse effects on development and health (11, 12).

The timing of brain development and associated neuroplasticity for motor skill learning makes early childhood a critical time for developing and reinforcing movement skills. Children who do not participate regularly in movement skill–enriched activities may never reach their potential for motor control, which underlies sustainable PA and physical fitness later in life (13). The mastery of fundamental movement skills (FMS) can be regarded as a building block (14), which is important for children's development (15) and for learning more complex skills. Intervention studies have shown that lessons targeting FMS development lead to higher PA levels, while structured PA leads to better mastery of FMS (16, 17). Thus, there is a reciprocal relationship (18–21).

As most children attend ECEC, it is an important arena for developing adequate FMS (22, 23) as well as for raising PA levels among young children (21). Educators need to know how to establish conditions for children to learn MoPA and to make decisions on what and how to teach (24). In addition, educators' attitudes toward MoPA, being outdoors with the children, participating in MoPA themselves were of great importance for children's opportunities for doing MoPA in ECEC (8, 25, 26). The educators' didactic skills, to know what to teach and to know how to teach it are vital for the outcome. Three components of educators' knowledge required for teaching were identified: content knowledge (CK), pedagogical knowledge (PK) and the combination of the two, pedagogical content knowledge (PCK) (27). Teacher's teaching competence consists not only of the specific CK but also of knowledge about students' learning within the subject and requires knowledge about governing documents, about the purpose and context of education, as well as general PK and didactic skills (28). The combination, PCK, influences teaching in ways that best engender children's learning for understanding the content of different domains. In this study, the domain of MoPA is in focus.

The perspective of metacognition is described as the higher-order thinking that involves active control over the cognitive processes engaged in learning and is the understanding of one's own thoughts and knowledge (29). Metacognition is affected by internal factors such as critical thinking and learning strategies (which are conscious processes) but also by unconscious processes such as motivation and attitudes. External factors such as education, workplace actions and projects, and familial factors have been shown to affect metacognition (30).

Studies on ECEC educators' perceptions and experiences about MoPA are scarce in Sweden. Given the large number of children aged one to five years spending a significant amount of time in ECEC, the purpose of the action-oriented study was to get an insight into the everyday life in the Swedish ECEC and understand the educators' reasoning and descriptions of how they handle the teaching of MoPA. The specific aims were to explore ECEC educators' perceptions and experiences of planning and performing MoPA sessions among children, and to see if and how the educators' perceptions and discussions in the focus groups changed during the study period of 18 months.

## 2. Materials and methods

### 2.1. Participants

In Sweden, ECEC employees are either preschool teachers or day-care attendants. About half of all ECEC employees are preschool teachers who have three and a half years of university training; the other half are day-care attendants who have upper-secondary qualifications (31). In this study, all the employees are named educators and the two groups are not separated. They were mixed in the focus groups.

The participants were 88 ECEC educators, preschool teachers (36), and day-care attendants (52) (aged 20–65 years old) working in five preschools in municipalities with 10,000 to 95,000 inhabitants in southern Sweden. The sampling was done in two steps. In the first step, preschools were selected focusing on the participatory action-based design, where collaboration and active participation among the participants were important for the research process (32). The five selected preschools represented both the private and public sector. In the second step, all the educators at these five preschools were invited to participate. Informed consent was obtained from all the educators, no one declined.

During the 18 months project, all MoPA activities among children were planned and carried out by the educators in their ordinary work. They were asked to film self-selected MoPA sessions. The filmed sequences were later shown in the focus group meetings and were the starting point for the discussions. The MoPA session situations in the preschools were supposed to be as like the ordinary workday as possible to see what knowledge and routines existed in the staff groups and what was offered to the children. The project focused on MoPA but did not involve education *per se* for the staff.

### 2.2. Methods

A qualitative research approach with focus group discussions was chosen due to the aim of exploring educators' perceptions and experiences of MoPA. It was supplemented with filmed sequences from the MoPA sessions in the preschool, which could be seen as observational fragments. The focus group discussions were implemented in an informal conversational manner, led by the same moderator in all focus groups. The filmed sequences from the work with the children illustrated educators and children in MoPA sessions and initiated the collegial discussions. The focus group discussions made it possible for the moderator to gain insights into complex interrelations within the groups (33, 34) and the ways the discussions developed over time. The filmed sequences made it possible for the researchers to gain insights into the planned and implemented activities, to follow how the educators acted among the children and how the MoPA activities were discussed in the focus groups. The educators received small logbooks that they could keep in their pockets to make short and informal notes about MoPA in their working day. The logbooks were not collected afterwards. They were only used by the educators as their own memory support. There were no ready-made questions for the discussions in the focus groups; issues arising from the filmed sequences and the participants' experiences and thoughts formed the basis of the discussions. Each focus group at each of the five preschools met on six occasions over a period of 18 months. Each focus group comprised ten to 14 participants, and each discussion lasted one to one and a half hours. Throughout the project the educators planned and carried out all MoPA activities on their own without any kind of intervention from the research team. A work situation that was as normal as possible, where educators' existing competence and routines were used was sought.

### 2.3. Analysis

The focus group discussions were recorded and transcribed verbatim. A content and thematic analysis inspired by Graneheim and Lundman (35) (2004) and (36) was undertaken to identify perceptions and experiences of MoPA, with help from the research questions:

(1) How do educators perceive and experience teaching in MoPA?
(2) How do educators perceive the role of MoPA in ECEC?

A perspective of PCK, as described by Shulman and Shulman (1987, 2009) was used as the point of departure in this study, as well as the perspective of metacognition, as described by Dunlosky and Metcalfe (2008). The analysis of the transcribed material from the focus group discussions was supported by six steps: familiarization, coding, generating themes, reviewing themes, defining and naming themes, and presentation of findings (35, 36). Familiarization included listening to the recorded discussions and reading them aloud back and forth during the transcription process. Coding and generating themes were done by identifying a set of statements (meaningful units), relevant to the research questions. The meaningful units were condensed and coded into themes and subthemes (35).

### 2.4. Ethics

The study was conducted in accordance with the ethical principles for research involving human subjects, and all procedures were in accordance with the Declaration of Helsinki and the Swedish law on research ethics (SFS:2003:460). An ethical review application was approved by the Regional Ethical Review Committee in Lund (Dnr: 2017/555). The participants were informed about the study, their voluntary status, and confidentiality. Written informed consent was obtained from all participants.

## 3. Results

The analysis revealed three themes —*Teaching aspects; Educational aspects; Organizational aspects* —with associated subthemes, which are presented in Table 1 along with examples of condensed meaningful units (see Table 1).

**Table 1:** Themes with associated subthemes identified in ECEC educators’ focus group discussions, as well as examples of condensed meaningful units.

Theme and subthemes	Examples of condensed meaningful units
Theme 1. Teaching aspects	
a) Competence	Own physical capacity and fitness
	Select right or wrong activities
	Leadership of mobile children
	A wish to guarantee safety
b) Role modeling	Own physical capacity and fitness
	Self-confidence and priority of domains
	Easy to fall back to lethargy
Theme 2. Educational aspects	
a) Children's development	Many domains to develop, MoPA low priority
	MoPA for other goals, not for its own sake
	Compensatory mission for ECEC
	School readiness skills
b) Children's health and wellbeing	Health determinants
	Group vs. individual observations
	Childhood vs. other phases in life
Theme 3. Organizational aspects	
a) Curriculum	Vague guidance, low priority for MoPA
	Stress achieving academic skills
	Unspoken demands
b) Environment	Indoors – several restrictions, sedentary
	Outdoors – free play, some restrictions
	Colleagues’ influences and demands
	Parents’ influences and demands
	Managers’ and administrators’ demands

### 3.1. Teaching aspects

#### 3.1.1. Competence

The discussions indicated that the educators perceived that their knowledge about what to teach and how to teach MoPA was inadequate. They perceived that it was difficult to select relevant MoPA activities and they worried whether the activities were “right”. The variety of movements was therefore limited, and the content in the sessions was often the same irrespective of the children's age or skills. They found that it was especially difficult to select exercises to challenge the children to make progress. The low CK and lack of progression knowledge strongly limited the planning of the MoPA sessions. The educators emphasized that the main objective of the activities was to let the children feel joy and happiness. Therefore, the activities were separate ones selected for pleasure and not for learning skills. It was stated that it was important that all children should be able to perform the activities without any difficulties, and therefore the level of challenge was low. Many of the educators said they were afraid of teaching MoPA as it could be interpreted as boring, and they selected only well-known and popular activities. Structured MoPA teaching was seldom performed. Most sessions consisted of a short play, led by the educator, followed by free play. The educators hoped that the children would learn enough MoPA skills through the free play.

The view on MoPA during free play changed during the project. In the beginning, the educators said that the children's MoPA levels were high during free play. But as the research project progressed, the educators became increasingly aware of the children's different activity levels, and gradually they observed the children individually. They observed that some children were sedentary and inactive most of the time in free play, which was a new insight for them. They said they became aware of the children's different FMS and MoPA and discussed how they could involve inactive children. At the end of the project, the educators wanted to plan more challenging activities for the inactive and active children. However, they perceived that they focused greatly on safety, which hindered the challenging effects. The educators said that they discovered that especially the challenges for the older or more skilled children were scarce as the activities were the same for all. The number of planned MoPA sessions increased during the project, but the educators perceived that they lacked competence to vary activities and challenge the children and found this a troublesome barrier. The educators' discussions were substantially more detailed and insightful at the end of the project regarding both the selected activities and the children's MoPA skills. Notably, the more insightfully they spoke about the children's MoPA skills, the more they argued that their own knowledge to teach MoPA was insufficient.

Several of the educators concluded that they were afraid of doing harm or causing injuries if they selected “wrong” activities or if the activity tempo was high. Besides difficulties in selecting what to teach, they perceived knowing how to teach as even more difficult. The educators emphasized the difficulties of being responsible for a group of mobile children, and on top of it giving instructions, observing each child's performance and giving feedback. Most of them found this unattainable. Those few who were confident in the MoPA domain were sport trainers in their leisure time. Most of the educators said they preferred teaching sedentary domains, such as language and esthetic activities. Teaching MoPA in a group of children was beyond their pedagogical competence and leadership skills. They were afraid of not being able to guarantee the safety of the children. Many of the educators found it quite frightening to let children move at high speed and tried to avoid it or slowed down the children. In addition, some educators revealed that their own fitness was low, and they were to a great extent unable to do MoPA themselves. Personal physical status was discussed, and the educators perceived that their personal fitness was important for self-confidence to lead MoPA, but most of those with low fitness were not ready to start their own training to increase fitness and to become more confident. However, some educators reported that they had become more physically active in their leisure time, which in turn affected their view on MoPA among children. The educators discussed the low MoPA levels among not only preschool-aged children but also among adolescents and adults in general. Those who said they had positive attitudes toward PA and were physically active said they gave MoPA high priority among children. At the beginning of the project, they were cautious about verbalizing this publicly to the group, given the assumption of a strong unspoken opinion by other group members that academic skills should be prioritized. At the end of the project, there was a shift toward more positive attitudes to MoPA in the groups, and the physically active educators were valued as resources and their advice and ideas were solicited.

#### 3.1.2. Role modeling

Role modeling was perceived as very important for children's learning. The motto “children do not do as the adults say but do as they do” was mentioned several times. The educators perceived they were aware of the importance of being good role models for the children, but many expressed difficulties in living up to the task when it came to MoPA. Some thought that role modeling was more connected to attitudes than to knowledge and that they could promote MoPA without CK, while others thought this was impossible. The fear of injury during MoPA was pervasive and was reported to hold the educators back from doing more MoPA with the children. They feared injuries among the children, but many of them were also afraid of getting injured themselves. The preschools had many restrictions prohibiting movement to minimize injuries and some of the educators noted that it could be difficult to serve as an active role model in MoPA while following all the actual restrictions and policies.

Many of the educators perceived MoPA as strenuous to perform, and many failed to do MoPA themselves with the children even if they could. Most of the educators perceived that MoPA was the hardest area to fulfill in model learning. It was common to use purchased ready-made activity programs shown digitally on a screen. The educators found this convenient and trusted that the programs were appropriate, and right for children of different ages. The educators said they sometimes participated in the purchased programs with the children, but usually they relied on instructions from the speaker and did not participate.

### 3.2. Educational aspects

#### 3.2.1. Children's development

The educators argued that MoPA was valuable in a variety of developmental areas, for example for academic and cognitive performance. When development of MoPA for its own sake was discussed, the discussion was perceived as difficult, and the educators said they had insufficient knowledge about this developmental domain. Overall, MoPA development was perceived as complicated, and many educators referred frequently to children's “prerequisites”. They could not clearly define what they meant by “prerequisites”. The educators often mentioned that some parents always carried the children or pushed them in prams, and they perceived these children's motor development as delayed. When asked how they worked to compensate for these delays, they said that they did not know how to do it. The discussion about children's physical development and MoPA skills was characterized by uncertainty and avoidance among the educators.

The role of the ECEC in compensating for deficiencies in the children's “prerequisites”, such as low levels of MoPA at home which affected physical development, was discussed. Physical development could be affected by nutrition in ECEC, but the educators were doubtful whether they adequately could compensate for home situations when it came to MoPA. The educators thought of the compensatory role of ECEC when it came to languages and mathematics, but not in MoPA. Learning FMS and specific MoPA skills was, to a great extent, an undisputed area. The educators described learning in terms of academic skills such as language, science, and mathematics rather than MoPA skills. Skills for school readiness were discussed and the educators had difficulty finding any MoPA skills required for school readiness. The ability to walk, dress, and eat were suggested as important MoPA skills, but not skills such as running, jumping, performing handstands, somersaults, etc. The educators reported they could not teach specific MoPA skills, and they said they were happy to be able to walk with the children and let them run and climb freely in the natural surroundings in free play. Some educators endorsed that FMS and other MoPA skills should take place only during free play and should not be formally taught at all, while many educators expressed uncertainty on how best to provide instructions and organize appropriate learning opportunities, describing this uncertainty as a barrier. They wanted further education to be capable to teach children MoPA skills.

#### 3.2.2. Children's health and wellbeing

When discussing MoPA for health, all the educators argued for its benefits, and most of them found it beneficial. Focus in the discussions was also put on how much MoPA is necessary to maintain and promote good health. The discussions about the moderate-to-vigorous-physical-activity (MVPA) recommendations from the World Health Organization (WHO) (37) engaged the educators, who said they perceived it was difficult to reach the recommended MVPA in ECEC. On the other hand, they contended that the children were in constant motion, suggesting that the children exceeded the WHO recommendations. The reason for this discrepancy in perceptions remains unclear, but the educators said they mostly observed children in groups, which could affect the view that children move constantly. Gradually during the project, the educators observed the children individually rather than in groups, and some were surprised at how inactive and sedentary some children were during a day at preschool. Although the educators argued for the health benefits of MoPA, they were not ready to promote a substantial increase of MoPA in ECEC. They discussed how they could increase children's time running outdoors, which they found the easiest way to increase MoPA for health. The increase of children's movement skills was seen as more difficult to realize as they did not know how they could teach it.

### 3.3. Structural aspects

#### 3.3.1. Curriculum

The discussions in the focus groups revealed that the national policy documents were perceived as simultaneously vague and too extensive to be manageable. The educators perceived that MoPA was only briefly addressed in the national curriculum. Therefore, little attention was paid to it either in actions or in references. The educators often reiterated the limited time allocations provided to meet the fully packed curriculum. An unspoken priority list of activities was found to exist in the daily work. The educators could not explain why but said the curriculum prioritized achieving goals in academic and social domains over those in MoPA, and therefore more time was allocated to them. On one hand they wanted a more distinct curriculum, which clearly described what to do with the children, but on the other hand they did not want to be forced to do MoPA with the children. The conclusion at the end of the discussion was that they preferred freedom in doing what they wanted when it came to MoPA.

#### 3.3.2. Environment

The physical environment was highlighted as important for the promotion of MoPA. Although most of the educators were satisfied with the indoor and outdoor facilities at their preschool, they pointed out that the indoor environment was not designed for MoPA. There were several movement restrictions, and in most preschools, it was totally forbidden to jump or run indoors. Most of the educators did not use the indoor facilities for teaching MoPA. During the project, one preschool started to implement MoPA sessions before lunch indoors.

The outdoor environment offered more space and could be used for MoPA. However, as the climate in Sweden is quite cold, for several months a year the children are wrapped up in warm clothes, which constricts their movements. The outdoor environment was predominantly used for free play and not structured teaching in MoPA. Overall, teacher-led activities were rarely reported, either indoors or outdoors, and the educators said that they did not have enough space or tools for MoPA, though they said that they were satisfied with the environment. During the project, some educators reported using the actual spaces more regularly and made efforts to use both the indoor and outdoor environment for MoPA. One preschool was able to hire a gym hall and arranged MoPA for the oldest children once a week. Other educators focused on how to use the outdoor environment in challenging ways, such as exploiting differences in terrain height during teacher-led activities. The educators who accomplished MoPA actions were surprised at the rapid and unexpected progress made by the children. They reported that these outcomes had encouraged them to improve the teaching and the use of the environment.

When the MoPA project started, the educators had support from both parents and school administrators to increase daily MoPA. They were surprised at the support and wanted to meet the expectations. The educators partly did so, but they also perceived that they exposed the weaknesses in their competence for teaching MoPA. They perceived they quickly lost inventiveness regarding what to do in MoPA and they mostly did the same things. This became obvious through the filmed sequences. The project lasted for 18 months, and the same activities were repeated over and over again. The educators said they saw the monotony in the MoPA sessions and felt a need for further education.

## 4. Discussion

The findings in the participatory action-oriented research project with preschool educators will be discussed in relation to the research questions under the headlines: *Educators' perceptions and experiences of teaching MoPA; Educators’ perceptions of the role of MoPA in ECEC*.

### 4.1. Educators' perceptions and experiences of teaching MoPA

The findings of the study showed that most educators found MoPA difficult to teach. They preferred to teach more sedentary activities such as language, science or drawing, which is in line with other studies (4). Findings from a recent study indicated that MoPA, is a low-priority value, to varying degrees, in the ECEC curricula enacted by Nordic countries, especially in Sweden where the guidance provided to educators and stakeholders was inexplicit (38). The educators seldom or never taught structured MoPA and relied on the children's free play for physical and motor skill development. Most of the MoPA sessions consisted of a short teacher-led play, and free play the rest of the time. Studies have shown that children spend less than 50% of a free play period participating in MoPA (39). Providing children with structured MoPA could substantially increase the total amount of MoPA (40).

FMS are essential for present and future PA. Giving children possibilities to improve FMS and increase PA levels through adequate structured MoPA sessions is vital if they are to enjoy meaningful lives through play and engage in enjoyable MoPA in childhood but is also important from a long-term perspective (41, 42). In our study, it was found that educators perceived that their low PCK for teaching FMS and MoPA was a barrier to teaching adequate MoPA, which is a problem. It is important to develop FMS in early childhood (13, 15, 22) and structured MoPA can improve children's FMS (43). FMS are essential building blocks for learning more complex skills and for the ability to be physically active in childhood and later in life (18, 20, 21). The low priority of MoPA in ECEC (7, 8) and general low PA levels among children (9, 10) may have negative effects on children's actual and future health, as well as on public health. Early adoption of improved FMS and MoPA may mitigate the decline in PA often seen during the transition from childhood to adolescence (44) and further to adulthood (45). Fitness is a strong predictor of longevity and is inversely related to all-cause mortality (46). It should be noted that there is a heavy economic burden on health-care systems. Intervening early in life by promoting MoPA may not only help prevent chronic disease and provide cost savings for society, but it can also enrich children's pleasure in movement and general development. Health outcomes from early childhood are essential for sustainability (47, 48), and are included in the convention on the rights of the child (49).

ECEC is an ideal setting for increasing FMS and MoPA, given the large number of young children enrolled (50, 51). A thorough understanding of content is an essential prerequisite for teaching a topic, and CK is one of the central anchors of teachers' professional knowledge base (52). During early childhood, when most children attend ECEC in the Nordic countries, it is important to develop movement skills in the ECEC setting. The expectations on educators in ECEC are high, and the low PCK in MoPA is troublesome. The design of the actual project, in which the educators planned and performed activities among the children, where the outcomes were discussed afterwards among colleagues, forced them to use their PCK and reflect on the outcome. They perceived difficulties in varying the content of the MoPA sessions, which indicated that CK was low. They also perceived difficulties leading a group of mobile children indicating low PK. During the project they made progress, but still perceived low PCK. According to Shulman and Shulman (2009) teachers learn *via* critical reflections on their own practice and on the way, they transform their individual experiences into more generalizable conceptions *via* individual and collective reflection. Competence and CK fit well with the centrality of subject matter (28). Many educators in our study argued that they still preferred ready-made MoPA programs, which might indicate low CK. It could also indicate indolence as they thought it was too strenuous to plan their own programs. Most of the educators were not ready to leave their comfort zones and were not open to new ideas, extra work or effort. Both effort and moments of alienation in the form of cognitive dissonance are crucial for getting teachers out of their comfort zones (53). Personal PCK develops as a teacher makes individualized refinements to the practice (54).

The efforts may at first be perceived as insurmountable and too demanding. Initially, the educators seemed to rely on the PK, with CK becoming important in their practices over time. The educators in our study were challenged to develop their teaching and lessons, which challenged their PCK. They had to endeavor to reach new insights into how best to organize and implement appropriate MoPA in the preschool children's daily routines. They perceived they had to change their didactic methods. However, low CK was a great barrier and influenced the possibility of making big changes in PCK. They highlighted that they needed education in MoPA to learn more to be able to vary teaching and challenge the children's individual development. Experiences from the project increased the metacognition and made them observant of the problem, which made it easier for the educators to formulate their need for PCK and wish for further education.

### 4.2. Educators' perceptions of the role of MoPA in ECEC

Several reports have raised the issue of low prioritization of MoPA in ECEC (7, 8). At the beginning of the project, MoPA was given low priority in the preschools, but it gained more importance during the project. The project may have started the process of metacognition about MoPA among educators, which led to behavioral changes and new didactic methods for some. As the MoPA project lasted for 18 months, subconscious processes such as priming appear to have influence on the educators' behaviors and belief systems, thereby contributing to the development of metacognition. Priming is described as mental non-conscious processes activated by environmental stimuli (55). It is a phenomenon whereby exposure to a stimulus, in this case actions and collegial discussions about MoPA, influences a response to a subsequent stimulus without conscious guidance or intention (56). The influence from continuous meetings, i.e., a form of priming, led to increased metacognition, and for some educators also led to changed behavior. Both conscious and non-conscious processes contribute to increased metacognition (57). However, many educators did not leave their comfort zones, despite increased metacognition. Some were not ready to leave the comfort zone, though they would notice new opportunities, experiences and personal growth await if they could step across that line (58).

Individuals who value stretching themselves to try unfamiliar things are more confident that they can perform tasks that fall outside their comfort zone (59). The beliefs that people hold about their own capabilities are critical elements for their behavior. The ways people behave can often be better predicted by these so-called self-efficacy beliefs than by what they are capable of accomplishing (60). The educators' perceptions of the role of MoPA in ECEC were often predicted by self-efficacy issues. During the project the focus group discussions became more detailed and insightful about MoPA, which reflected efficacy and motivation (60, 61) as well as increased metacognition (57).

The educators perceived those supportive comments from colleagues in the focus group discussions influenced their efficacy and motivation. Trust in colleagues and collective teacher efficacy could significantly and positively account for the school-level variations in teachers' commitment to teaching a topic (62). In addition, the overwhelming support from the preschool children and the parents for the increased MoPA levels was also perceived as positive for the educators' creativity and efficacy. Creativity is positively associated with encouragement and motivation, highlighting the mediating role of creative process engagement in facilitating performance (63).

Although behavior and thoughts can be conceptualized as regulated by executive functions, subconscious processes play a crucial role in human behavior (57). Subconsciously offered attitudes appear to be strong driving forces in changing mindsets and behavior, sometimes more so than consciously offered attitudes (64). Subconscious processes activated by an environmental stimulus, together with conscious processes such as behavioral MoPA implementations, can increase the likelihood of conscious behavioral actions (65). In this project, the subconscious processes were very likely activated by the collegial discussions. Together with the conscious trial-and error activities in planning and performing MoPA sessions among children, the subconscious processes could have affected the educators’ metacognition and for some educators also affected behavioral actions. The educators who increased their leisure time PA, which in turn affected the priority of MoPA in the work with pre-schoolers, may have been affected by both conscious and non-conscious processes. The interactions between conscious and subconscious processes are complex and nuanced (66) and PA behavior is often influenced by implicit attitudes (67).

Unintended carryover effects from priming may have affected the educators' interest in MoPA and need to increase CK. Carryover effects predict the sensitivity to the content and are also mediated by teachers' self-efficacy beliefs (68). The educators in our study became more observant of children's different levels of MoPA during the project. In the beginning, many educators observed groups of children and concluded that children moved continuously. In the end, their observations focused more on individual children's MoPA, which revealed that many children were inactive. This insight seemed to be the starting point for increased metacognition. Many of the educators also became aware of their own PA levels and among people in general, which affected their perceptions of the role of MoPA in ECEC.

### 4.3. Strengths and limitations

The study has some limitations and strengths worthy of discussion. Overall, the design of qualitative research has methodological considerations. According to Nowell et al. (2017) (36) there are limitations with content and thematic analysis, but also strengths. The study focused on the educators' narratives of their perceptions and experiences, which limits the generalizability. It is vital to explore educators' perceptions of planning and performing MoPA sessions in their ordinary work, especially over a longer period, which was manageable in a participatory project with a qualitative approach. It was important to involve and work closely with the educators and they were involved in the design of the project, which could be seen as a limitation, but also as a strength as it gave the possibility to investigate experiences of ordinary practice. The participatory-oriented study may explore everyday work and give the employees voices about their perceptions and experiences.

Five preschools with 88 employed educators participated in the project. The sample could be seen as small from the perspective of statistics, but in this study with a qualitative approach it could be seen as adequate. It is the nature of exploratory qualitative research to adopt a narrow focus to obtain in-depth contextual data. The content analysis was carefully carried out and the study findings were checked by a selection of participating educators during and after the analysis. The study period of 18 months was substantial and provided opportunities to follow changes in the collegial discussions and the educators’ personal reflections. The collegial discussions in the focus groups provided a safe forum and afforded closeness where the educators were able to discuss their experiences. Both preschool teachers and day-care attendants, in this study named “educators”, participated in the project as they worked with the children. They had different educational levels but were not separated in the study. If separated, the discussions might have been different. The research about priming and metacognition in the context of PA is limited (69), and to the best of our knowledge, no study to date has explored priming and metacognition in the context of teaching MoPA.

## 5. Conclusions

It can be concluded that continuous meetings and collegial discussions contributed to the ECEC educators’ enhanced metacognition about MoPA, which in turn highlighted low PCK and a need for improved education. Priming *via* a collegial focus on MoPA may start both non-conscious and conscious processes which may lead to increased metacognition and occasional behavioral changes, which in turn can provide a blueprint for implementation of MoPA among educators in ECEC. Additional structural processes such as collegial cooperation, administrative and parental support seem to enhance changes in preschool policy and the priority of MoPA and reinforce positive effects. During the trial-and-error MoPA teaching, the educators detected insufficient PCK to teach MoPA and that teaching was often replaced with free play. Increased metacognition made the educators aware of children's different MoPA levels and that free play did not always increase all children's skills. ECEC is an ideal setting for teaching FMS and MoPA, given the large number of young children enrolled. As high expectations are put on educators in ECEC for children's development, the perceived low PCK in MoPA is troublesome. MoPA levels among children are in general low, and negative effects on children's actual and future health may occur. Early adoption of improved MoPA and an active lifestyle may mitigate the decline in PA often seen during the transition from childhood to adolescence and further to adulthood. Although there was increased metacognition about MoPA among ECEC educators, the PCK was found to be insufficient to develop adequate teaching in MoPA and the study highlighted the need for improved education in MoPA in early childhood teacher education as well as among working professionals to enhance the PCK. Further studies on this topic are warranted.

## Data Availability

The datasets presented in this article are not readily available because of restrictions due to ethical reasons.Requests to access the datasets should be directed to annchristin.sollerhed@hkr.se.
